# Sertraline use in pregnancy and placental transfer: A pharmacokinetic prospective cohort study

**DOI:** 10.1002/pmf2.70093

**Published:** 2025-09-05

**Authors:** Charlotte Goutallier, Stephen Tong, Cassius Phogole, Tracy Kellermann, Manarangi De Silva, Eric Decloedt, Catherine A Cluver, Roxanne Hastie, Anthea Lindquist

**Affiliations:** ^1^ Mercy Hospital for Women Heidelberg Australia; ^2^ Department of Obstetrics and Gynaecology University of Melbourne Melbourne Australia; ^3^ Mercy Perinatal Mercy Hospital for Women Heidelberg Australia; ^4^ Division of Clinical Pharmacology Department of Medicine Stellenbosch University Cape Town South Africa; ^5^ Department of Obstetrics and Gynaecology Stellenbosch University and Tygerberg Hospital Cape Town South Africa

**Keywords:** anti‐depressant, anxiety, depression, pharmacokinetics, placental Transfer, pregnancy, sertraline, SSRI

## Abstract

**Introduction:**

Depression in pregnancy is common and the use of antidepressants in pregnancy, especially sertraline, is on the rise. Although sertraline is known to cross the placenta, the level of transfer to the foetus is unclear. Thus, we investigated maternal and umbilical cord blood sertraline levels following use in pregnancy.

**Methods:**

We prospectively recruited women taking sertraline during pregnancy, who were undergoing a caesarean section birth (*n* = 18). Maternal and umbilical cord blood samples were collected at the time of caesarean and sertraline and desmethyl sertraline concentrations measured via liquid chromatography with tandem mass spectrometry.

**Results:**

The ratio of umbilical cord to maternal plasma concentrations was calculated to determine placental transfer. Sertraline and desmethyl sertraline concentrations were consistently lower in umbilical cord blood than maternal plasma, with ratios of 0.35 for sertraline and 0.43 for desmethyl sertraline, suggesting incomplete placental transfer. Higher maternal doses and shorter intervals since the last dose correlated with higher foetal exposure. There were three preterm births (16.7%), and one baby with a major congenital abnormality (William's syndrome) among our cohort. Adverse neonatal outcomes were uncommon, with all term infants having Apgar scores > 5 at 1 and 5 min and 4 experiencing respiratory distress.

**Conclusion:**

Our findings suggest maternal use of sertraline in pregnancy results in moderate placental transfer and, thus, foetal exposure. Umbilical cord blood levels were influenced by maternal dose and timing of administration. These results may assist in shared decision‐making for clinicians and patients when considering the initiation or continuation of psychotropic treatment in pregnancy.

## 1 INTRODUCTION

Depressive symptoms and major depressive disorder are common in pregnancy and affect women worldwide. Reports of prevalence vary widely from 8% to 51% [1, 2, 3] for depressive symptoms and from 10% to 17% [1, 4, 5, 6] for major depressive disorder. Selective serotonin reuptake inhibitors (SSRIs) are increasingly used to treat depression, with sertraline, the most commonly used SSRI during pregnancy, on the rise [7].

The short‐ and long‐term effects of most psychotropic drugs prescribed during pregnancy on exposed children are largely unknown [8]. The decision to continue or initiate psychotropic treatment during the periconceptual or antenatal periods poses a considerable challenge, driven by the need to balance potential risks to the foetus with minimising maternal morbidity.

Maternal risks of untreated depression during pregnancy include increased alcohol, tobacco and substance use, poor compliance with vitamin supplementation [9, 10], as well as suicidal ideation and suicide attempts [11]. Suicide is a leading cause of maternal death in the first year after childbirth, with studies indicating that it accounts for approximately 20% of postpartum deaths globally [12]. Untreated depression may also be associated with early pregnancy loss [13, 14], obstetric complications [15], preterm birth [14], small for gestational age and low birth weight [9, 15, 16, 17, 18] and neonatal care unit admission [19].

However, there is limited evidence about the safety of SSRI use during pregnancy. Some studies suggest that antidepressant use in pregnancy is associated with increased risk of early pregnancy loss [20], congenital abnormalities [21] and obstetric complications such as preeclampsia, postpartum haemorrhage [22, 23] and preterm birth [21]. There is evidence of risks to the neonate such as persistent pulmonary hypertension of the newborn, poor neonatal adaptation syndrome and stillbirth [21, 24, 25, 26, 27, 28, 29, 30].

Despite the importance of pharmacokinetic data to guide safe and effective dosing during pregnancy, existing studies are limited in scope and quality, often constrained by small sample sizes, missing data and inadequate information on timing and dosage [31, 32, 33, 34, 35, 36, 37, 38, 39]. These limitations are largely due to the practical and ethical challenges of conducting clinical studies in pregnant women, who are frequently excluded from pharmacokinetic research. Consequently, dosing recommendations for pregnant patients are often based on extrapolated data from nonpregnant populations, which may result in ineffective or unsafe dosing.

A 2023 literature review [40] on the pharmacokinetic changes in antidepressants during pregnancy identified only eight small studies focusing on sertraline, most of which highlighted the significant gaps in existing data. Addressing this shortfall, our study examines the placental transfer of sertraline and its active metabolite, desmethyl sertraline, in a larger cohort using a novel technique, advanced liquid chromatography with tandem mass spectrometry. By incorporating detailed maternal demographic and neonatal outcome data, our study offers insight into perinatal outcomes among neonates exposed to sertraline.

## 2 MATERIALS AND METHODS

### 2.1 Study population

The study cohort was recruited prospectively between June 2019 and April 2021. Patients were included if they had a singleton pregnancy, were undergoing caesarean section birth and were known to be taking sertraline during pregnancy. The timing of sertraline commencement, the dose and interval between the last dose and birth were documented.

### 2.2 Experimental design and analysis

Maternal plasma samples were obtained just prior to caesarean section. Umbilical cord blood was obtained from the umbilical vein after delivery of the baby but before separation of the placenta. Blood samples were collected in ethylenediamine tetraacetic acid (EDTA) tubes with the plasma fraction collected after processing. Samples were stored at −80°C until they were sent to the Division of Clinical Pharmacology, Stellenbosch University, South Africa, for pharmacokinetic analysis.

The method of plasma analysis has been described previously [41]. In brief, analytes were extracted from 200 µL of plasma by protein precipitation. Sample analysis was performed on a Shimadzu 8040 triple quadrupole mass spectrometer in the positive ionization mode, monitoring the transitions of *m*/*z* 306.1 → 159.1, 309.1 → 275.2, 292.1 →159.1 and 296.2 → 279.0 for sertraline, sertraline‐d3, *N*‐desmethylsertraline and *N*‐desmethylsertraline‐d4, respectively. Chromatography was achieved using a Poroshell EC‐C18 column (3 × 100 mm, 2.7 µm) and 0.1% formic acid in water (mobile phase A) and acetonitrile (mobile phase B) in gradient elution at a flow rate of 0.450 mL/min. The calibration ranges for sertraline and *N*‐desmethyl sertraline were 2.50–320 and 10.0–1280 ng/mL. The method met the validation criteria specified by the United States Federal Drug Administration (FDA and European Medicines Agency (EMA) guidelines [42, 43].

Concentrations of sertraline and its active metabolite, desmethyl sertraline, were measured in both maternal blood and umbilical cord blood. The lowest limits of quantification were defined as 2.5 ng/mL for sertraline and 10.0 ng/mL for desmethyl sertraline [41]. The ratio of both sertraline and desmethyl sertraline in umbilical cord blood to maternal plasma was calculated to reflect the degree of placental passage. A ratio of 1.0 suggests complete transfer across the placenta and a ratio < 1.0 incomplete placental transfer.

### 2.3 Data and statistical analysis

Individual patient records were reviewed to ascertain maternal and neonatal demographic and outcome data. Neonatal outcome data were obtained from medical records. Demographic and clinical characteristics of study participants and their neonates were described using median and interquartile range (IQR) or mean and standard deviation (SD), depending on the data distribution.

## 3 RESULTS

### 3.1 Study population

The cohort included 18 pregnant participants ranging in age from 26 to 40 years (median age 33 years; IQR 30.3–34.8) (Table 1). The median body mass index (BMI) amongst the cohort was 26 (IQR 22.5–35.2) at booking and 30 (IQR 29.0–36.8) at the time of birth. Most women (12/18; 66.7%) were multiparous and born in Australia (16/18; 88.9%).

**TABLE 1:** Maternal demographics.

Patient	Age (years)	BMI at booking (kg/m^2^)	Parity^a^	Pre‐gestational medical disorders	Gestational disorders	Mental health diagnoses	Indication for treatment	Commencement of treatment	Dose at time of delivery (mg/day)	Time since last dose (hours)
1	34	24	3	Nil ex‐smoker (ceased pre‐pregnancy)	Nil	Anxiety Depression	Anxiety Depression	Pre‐pregnancy	100	44–52
2	31	21	1	Nil	Nil	Anxiety Depression	Anxiety Depression	Pre‐pregnancy	150	20–28
3	30	22	1	Myasthenia gravis Dressler's syndrome	Nil	Anxiety Depression Post traumatic stress disorder	Anxiety Depression	Pre‐pregnancy	100	1–10
4	33	24	2	Nil	Nil	Anxiety Depression Post‐natal depression Panic disorder Post traumatic stress disorder	Anxiety Depression	1st trimester	200	0.5–9
5	35	36	3	Obesity ex‐smoker (ceased 18 years prior to delivery)	Cholestasis of pregnancy	Anxiety Depression	Anxiety Depression	Pre‐pregnancy	100	9–19
6	33	26	4	Previous gastric sleeve endometriosis ex‐smoker (ceased at conception, prior to this smoking for 18 years)	Nil	Anxiety Depression Bulimia nervosa in past	Anxiety Depression	1st trimester	200	20–28
7	30	43	5	Essential hypertension obesity smoker (3 pack years)	Preeclampsia	Anxiety Depression Post traumatic stress disorder	Anxiety Depression	Pre‐pregnancy	100	48–56
8	31	46	2	Obesity smoker (2 pack years)	GDM insulin	Depression	Depression	Pre‐pregnancy	100	36–44
9	28	37	2	Obesity serous ovarian tumour endometriosis	GDM diet Preeclampsia	Anxiety Depression	Anxiety Depression	1st trimester	100	11–19
10	34	23	1	Nil	Nil	Anxiety	Anxiety	Pre‐pregnancy	50	0.5–9
11	33	26	1	Asthma PCOS Endometriosis Gastric sleeve ex‐smoker and drug use (ceased cigarettes and marijuana at time of conception)	Nil	Anxiety Depression Post traumatic stress disorder	Anxiety Depression	Pre‐pregnancy	100	10–19
12	35	26	4	Nil	GDM diet	Anxiety Depression	Anxiety Depression	Pre‐pregnancy	100	1–9
13	26	22	1	Smoker (3 pack years)	Nil	Anxiety Depression Borderline personality disorder	Anxiety Depression	2nd trimester	100	21–29
14	35	36	3	Nil	Post partum preeclampsia	Depression Obsessive‐compulsive disorder	Depression	Pre‐pregnancy	300	8.5–17
15	38	22	1	Endometriosis	Nil	Anxiety	Anxiety	Pre‐pregnancy	100	0.5–9
16	31	21	3	Nil	Nil	Anxiety Depression Anorexia as teenager	Anxiety Depression	Pre‐pregnancy	100	2–11
17	27	34	3	Asthma obesity	GDM insulin Pregnancy induced hypertension	Anxiety Depression	Anxiety Depression	Pre‐pregnancy	50	9–17
18	40	33	2	Essential hypertension ex‐smoker (unclear when ceased)	Nil	Anxiety Depression	Anxiety Depression	Pre‐pregnancy	100	0.5–9

^a^ Parity post‐index pregnancy.

Several participants had documented pre‐gestational medical disorders, including obesity (*n* = 5; 27.8%), prior gastric sleeve surgery (*n* = 2; 11.1%) and essential hypertension (*n* = 2; 11.1%). Two women developed preeclampsia during pregnancy (11.1%) and four developed gestational diabetes (22.2%; diet‐controlled *n* = 2 and insulin‐treated *n* = 2). A history of smoking pre‐pregnancy was reported by five participants (27.8%), with a further three reporting ongoing smoking during the pregnancy (16.7%).

### 3.2 Indication for sertraline use in pregnancy

Anxiety and/or depression were the primary indications for sertraline use in pregnancy for all participants. Sertraline was prescribed by a general practitioner for 8 (44.4%) and a psychiatrist for 6 participants (33.3%); the prescriber was undocumented for four participants. A total of 14 patients commenced sertraline pre‐pregnancy, 71.4% (*n* = 10) at a dose of 100 mg daily, and the remaining four at doses between 50 and 300 mg. Sertraline was first initiated during pregnancy for a further 4 participants (22.2%), largely during the first trimester (3/4; 75%). Co‐morbid mental health conditions were documented amongst 8 women (44.4%) and included post‐traumatic stress disorder (PTSD) (*n* = 4), panic disorder (*n* = 1), bulimia nervosa (*n* = 1), borderline personality disorder (BPD) (*n* = 1), obsessive‐compulsive disorder (OCD) (*n* = 1) and anorexia nervosa (*n* = 1).

### 3.3 Sertraline concentrations

**FIGURE 1:**
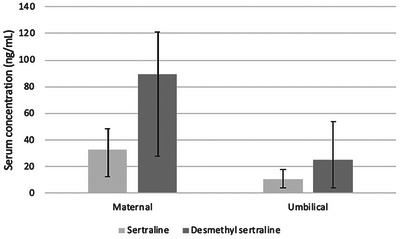
Median sertraline and desmethyl sertraline concentrations in maternal plasma and umbilical cord plasma samples.

Among the 18 maternal samples obtained, the median maternal plasma sertraline concentration was 32.7 ng/mL (IQR = 36.4; Figure 1). Values ranged from below the lowest limit of quantification of 2.5 to 120 ng/mL (Table S1). The median desmethyl sertraline concentration was 89.6 ng/mL (IQR = 93.7; Figure 1), ranging from below the lowest limit of quantification of 10.0 to 237 ng/mL. In umbilical cord plasma, the median sertraline concentration was 10.9 ng/mL (IQR = 13.6), with a range of below the lowest limit of quantification to 65.5 ng/mL (Table S1). The median cord desmethyl sertraline concentration was 25.4 ng/mL (IQR = 49.8), ranging from below the lowest limit of quantification to 183 ng/mL.

The ratio of umbilical cord blood to maternal plasma concentrations for sertraline ranged from 0.21 to 0.60, with a median of 0.3 (IQR = 0.1). For desmethyl sertraline, the median transfer ratio was 0.4 (IQR = 0.1), ranging from 0.22 to 0.82. The highest ratios of transfer of sertraline (0.60) and desmethyl sertraline (0.82) were observed in a participant taking sertraline at a dose of 200 mg daily, with the last dose administered within 9 h of sampling. The lowest transfer ratios of sertraline (0.21) and desmethyl sertraline (0.22) were observed in a participant taking 100 mg sertraline with the last dose 11–19 h prior to sampling.

### 3.4 Clinical outcomes

All 18 study participants gave birth to a singleton liveborn baby. As per the study design, all births were via caesarean section, with 16 elective and 2 emergency. Emergency caesarean was performed for preeclampsia and suspected intrauterine growth restriction (IUGR) (*n* = 2). Gestational age at birth ranged from 30+4 to 39+2 with three preterm births (16.7%). The median term gestational age (*n* = 15) was 38+3, with a mean term birthweight of 3343 g (SD = 475 g) (Table 2).

**TABLE 2:** Neonatal outcomes.

Patient	Gestational age at birth (weeks)	Birthweight (g)	Apgars (1,5 min)	Admission to SCN or NICU > 48 h, duration	Other perinatal morbidity
1	38+5	3330	9,9	No	Jaundice
2	38+1	4040	6,7	No	Nil
3	38+6	3530	9,9	No	Transient tachypnoea of newborn Jaundice
4	38+4	3470	9,10	No	Hypernatremia
5	38+0	2780	9,9	No	Nil
6	37+3	3124	7,9	No	Transient tachypnoea of newborn
7	30+4	1199	7,9	Yes, 55 days	William's syndrome with mild‐moderate pulmonary vein stenosis and hypoplasia of aortic arch Respiratory distress syndrome Late onset sepsis (*S. haemolyticus* and *S. epidermidis*) Hypoglycaemia Jaundice requiring phototherapy
8	38+2	4160	9,10	No	Nil
9	32+3	1258	6,8	Yes, 37 days	Respiratory distress (unspecified cause) Hypoglycaemia
10	38+5	3430	7,9	No	Hypoglycaemia
11	39+0	3320	9,9	No	Slow weight gain
12	38+0	3700	6,6	No	Hypoglycaemia Respiratory distress (unspecified cause)
13	38+1	2250	9,9	No	Nil
14	39+0	3550	9,9	No	Nil
15	39+2	3090	9,9	No	Nil
16	37+5	2783	8,9	Yes, 5 days	Respiratory distress syndrome Pneumothorax Jaundice Soft systolic murmur
17	38+3	3590	9,9	No	Hypoglycaemia
18	36+0	2668	8,8	No	Respiratory distress (unspecified cause)

All three preterm births in our cohort experienced respiratory distress at birth. One neonate was diagnosed with respiratory distress syndrome and the remaining two with an unspecified cause of respiratory distress. Two preterm neonates born at 30+4 and 32+3 weeks’ gestation required 55‐ and 37‐day admissions, respectively, to the Special Care Nursery (SCN) and Neonatal Intensive Care Unit (NICU). The neonate born at 30+4 weeks’ gestation was the only case of congenital anomaly identified—Williams’ Syndrome (*n* = 1). Amongst our preterm cohort, two neonates (66.7%) experienced hypoglycaemia and one neonate (33.3%) experienced jaundice. Among the 15 term‐born neonates, Apgar scores were all > 5 at 1 and 5 min. Four term neonates experienced respiratory distress (26.7%), including RDS (*n* = 1), transient tachypnoea of the newborn (*n* = 2) and other unspecified causes of respiratory distress (*n* = 1). One term‐born neonate (6.7%) required a 5‐day admission to NICU for respiratory distress requiring intubation. Other complications at birth included hypoglycaemia (*n* = 3, 20.0%) and jaundice (*n* = 3, 20.0%; none requiring phototherapy).

## 4 DISCUSSION

Using novel pharmacokinetic techniques, our study analysed the maternal plasma and umbilical cord plasma concentrations of sertraline and its active metabolite desmethyl sertraline. We also examined neonatal outcomes amongst the study cohort. The concentrations of sertraline and desmethyl sertraline in the umbilical cord plasma reflect foetal plasma concentrations and were able to be measured in a non‐invasive manner.

Overall, our results demonstrate universally lower concentrations of sertraline and desmethyl sertraline in umbilical cord plasma than in maternal plasma. Maternal sertraline dose correlated with umbilical cord concentration of sertraline; as sertraline dose increased, the concentration in maternal plasma and umbilical cord plasma also increased. However, this relationship was not linear, suggesting that increasing maternal sertraline dose does not necessarily correspond to a comparable increase in foetal exposure. Shorter time frames since the last dose of sertraline also resulted in higher maternal and umbilical cord sertraline and desmethyl sertraline concentrations at the time of collection. These results underscore the variable placental transfer of sertraline and its metabolite to the foetus, influenced by factors such as dosage and timing of administration. Higher doses and shorter intervals between the last dose and birth of the foetus generally correlated with higher maternal and foetal plasma concentrations.

The mean proportion of transfer of sertraline from mother to foetus was 0.35, and of desmethyl sertraline was 0.43, suggesting moderate placental transfer. Previous studies examining cohorts ranging from 1 to 24 participants have described rates of sertraline transfer across the placenta between 0.27 and 0.7 [31, 32, 33, 34, 35, 36, 37, 38, 39]. Concentrations below the lowest limit of quantification (2.5 ng/mL for sertraline and 10.0 ng/mL for desmethyl sertraline) were observed in maternal samples associated with sertraline doses of 100 mg daily and 50 mg nocte, particularly at longer intervals since the last dose. Lower doses and longer intervals thus likely impact drug transfer across the placenta.

Our findings are descriptive and do not provide evidence about causality or safety. This study is subject to selection bias, with participants being women undergoing caesarean section who agreed to participate. Medication adherence was not verified by dispensing records; however, most women reported regular use. Additionally, the timing of the last sertraline dose prior to delivery was self‐reported and variable, limiting the precision of the pharmacokinetic analysis. Metabolic factors, which may influence drug level, were not assessed in our study. Neonatal outcomes have been included for completeness; however, our sample size is insufficient to ascertain associations between maternal sertraline use and neonatal outcomes. Furthermore, maternal factors including obesity, pre‐existing hypertension and smoking status may also influence pharmacokinetics and neonatal outcomes; however, our sample size was too small to assess or control for their impact.

There were few adverse neonatal outcomes among the 15 term‐born neonates in our cohort, with all Apgar scores > 5 at 1 and 5 min and one NICU admission. These findings contrast with a 2017 study by Hogue et al. that reported increased NICU admissions and lower 1‐min Apgar scores in term neonates with in utero SSRI exposure [44]. Hypoglycaemia at birth occurred in three term neonates in our cohort, two in the setting of maternal gestational diabetes, which is consistent with past studies that have reported an increase in neonatal hypoglycaemia with maternal antidepressant use antenatally [45].

The association between SSRI use and preterm birth has been previously reported, with a 2020 meta‐analysis of 23 cohort studies suggesting a significantly increased risk of preterm birth with maternal antidepressant use [46]. In our cohort of 18 pregnancies, there were three cases of preterm birth (16.7%) but all were iatrogenic, in the setting of obstetric complications. All three patients were taking 100 mg sertraline from early pregnancy.

There was one case of major congenital abnormality—William's syndrome—detected within our cohort. This autosomal dominant disorder is characterised by developmental delay, cardiovascular disease, connective tissue and endocrine abnormalities and feeding difficulties [47] and indeed postnatal follow‐up for this baby revealed mild coarctation of the aorta and mild bilateral branch pulmonary artery stenosis. Another term neonate, with RDS and a pneumothorax, was diagnosed with a soft systolic cardiac murmur auscultated loudest at the right sternal edge. Transthoracic echocardiogram performed on Day 4 of life showed findings suggestive of antenatal stress but no congenital cardiac malformation. A 2021 meta‐analysis [48] of 20 studies encompassing over 5 million pregnancies found that maternal sertraline use during the first trimester was associated with an increased risk of congenital heart defects. In a separate pooled analysis [49] of nine studies, the association between major congenital anomalies and maternal sertraline use antenatally was non‐significant, although sub‐analysis of maternal sertraline use at any time during the first trimester was associated with increased risk of cardiac septal defects, respiratory system defects, limb defects and clubfoot. Our case was diagnosed in the context of multiple associated maternal comorbidities including obesity (BMI 43), smoking, essential hypertension and subsequent preeclampsia. More research in large cohorts is needed to clarify the association between SSRI use and congenital anomalies.

### 4.1 Strengths and limitations

Our study is strengthened by its size and our use of a novel method to ascertain maternal plasma and cord blood drug concentrations. The method employed is more time efficient than high‐performance liquid chromatography (HPLC), is simple, highly sensitive and efficient for the simultaneous quantification of sertraline and desmethyl sertraline. The plasma extraction volume required is small (200 µL) and its calibration ranges covered the proposed therapeutic concentration range for both analytes, unlike other LC‐MS/MS methods [41]. To our knowledge, this is the largest single drug SSRI cohort to examine placental drug transfer wherein detailed maternal demographics and neonatal outcomes have also been described.

Among our cohort, there was wide variation in the lead health practitioner responsible for prescribing SSRIs. This is likely to have resulted in differences in the threshold for commencement or continuation of pharmacological treatment of anxiety and depression in pregnancy. However, the focus of our study was on plasma transfer of medication, and our findings are unlikely to have been impacted by possible differences in underlying disease severity. Additionally, we did not have access to a dispensing record and so were unable to measure adherence to pharmacological treatment. Moreover, during pregnancy, there is an upregulation of these enzymes which could affect our interpretation of these results [50].

Due to the limited patients included, a wide range of self‐reported timing between last sertraline dose and sample collection, formal statistical comparison of sertraline dose and timing and foetal concentrations was not feasible. Future studies with standardised dosing intervals are needed to clarify these relationships.

## 5 CONCLUSION

Using novel methods and the largest cohort to date, we have confirmed that sertraline and desmethyl sertraline cross the placenta, albeit in lower concentrations than maternal plasma concentrations, with variability influenced by factors such as maternal dose and timing since the last dose. Our data do not suggest that foetal exposure is proportional to maternal dosage, which may have implications for medication commencement and dose adjustment in pregnancy. While most infants in our cohort experienced largely reassuring outcomes, our findings are descriptive and do not reflect medication safety. Further research into the perinatal and long‐term neurodevelopmental outcomes is crucial to ensure safe prescribing practices, better inform clinical decision‐making and ensure optimal maternal and neonatal health outcomes.

## ETHICS STATEMENT

Ethical approval was obtained through the Human Research Ethics Committee of the Mercy Hospital for Women (R11/34). Eligible women were identified by a trained research midwife. Written informed consent was obtained for the collection of all samples and clinical data.

## DISCLOSURE

The authors report no financial or industry disclosures.

## Supporting information



Supporting Information
