# Supplementary material for: Sertraline use in pregnancy and placental transfer: A pharmacokinetic prospective cohort study
Source: Pregnancy (Hoboken). 2025 Sep 5;1(5):e70093. doi: 10.1002/pmf2.70093 (PMC13344681; doi:10.1002/pmf2.70093)
Supplement: Supplementary file 1 [file PMF2-1-e70093-s001.docx]

| Sertraline dose (mg/day) | Time  since last dose (hours) | Maternal Plasma | | Umbilical Cord Plasma | | Ratio of umbilical cord to maternal serum | |
| --- | --- | --- | --- | --- | --- | --- | --- |
|  |  | **Sertraline (ng/mL)** | **Desmethyl  sertraline (ng/mL)** | **Sertraline (ng/mL)** | **Desmethyl  sertraline (ng/mL)** | **Sertraline** | **Desmethyl  sertraline** |
| 100 | 44-52 | 13.9 | 88.6 | 3.61 | 21.0 | 0.26 | 0.24 |
| 150 | 20-28 | 33.3 | 123 | 13.6 | 56.6 | 0.41 | 0.46 |
| 100 | 1-10 | 57.9 | 95.6 | 24.6 | 54.9 | 0.42 | 0.57 |
| 200 | 0.5-9 | 110 | 225 | 65.5 | 183 | 0.60 | 0.82 |
| 100 | 9-17 | 49.0 | 129 | 18.5 | 55.4 | 0.38 | 0.43 |
| 200 | 20-28 | 32.1 | 50.7 | 12.3 | 22.3 | 0.38 | 0.44 |
| 100 | 48-56 | 4.92 | BLQ | BLQ | BLQ | - | - |
| 100 | 36-44 | BLQ | BLQ | BLQ | BLQ | - | - |
| 100 | 11-19 | 32.6 | 115 | 6.72 | 25.8 | 0.21 | 0.22 |
| 50 | 0.5-9 | 11.9 | 24.4 | 5.88 | 15.6 | 0.49 | 0.64 |
| 100 | 10-19 | 38.8 | 90.5 | 11.2 | 25.0 | 0.29 | 0.28 |
| 100 | 1-9 | 32.8 | 60.9 | 10.6 | 25.8 | 0.32 | 0.42 |
| 100 | 21-29 | BLQ | BLQ | BLQ | BLQ | - | - |
| 300 | 8.5-17 | 120 | 237 | 31.8 | 82.4 | 0.27 | 0.35 |
| 100 | 0.5-9 | 19.6 | 37.1 | 5.16 | 0.00 | 0.26 | 0.00 |
| 100 | 2-11 | 73.3 | 116 | 26.6 | 50.2 | 0.36 | 0.43 |
| 50 | 9-17 | BLQ | BLQ | BLQ | BLQ | - | - |
| 100 | 0.5-9 | 48.0 | 128 | 14.9 | 44.3 | 0.31 | 0.34 |

Supplemental Table 1: Individual sertraline and demethyl sertraline concentrations for each participant. BLQ=below level of quantification.
